# Factor structure of the General Health Questionnaire-28 (GHQ-28) from infertile women attending the Yazd Research and Clinical Center for Infertility

**Published:** 2015-12

**Authors:** Zahra Shayan, Zahra Pourmovahed, Fatemeh Najafipour, Ali Mohammad Abdoli, Fatemeh Mohebpour, Sedighe Najafipour

**Affiliations:** 1 *Department of Community Medicine, Medical School, Shiraz University of Medical Sciences, Shiraz, Iran.*; 2 * Social Determinates of Health Research Center* *,* * Shahid Sadoughi University of Medical Sciences, Yazd, Iran.*; 3 *Shiraz University of Medical Sciences, Shiraz, Iran.*; 4 *Yazd Research and Clinical Center for Infertility, Shahid Sadoughi University of Medical Sciences, Yazd, Iran.*; 5 *Department of Medical Education, Medical School, Tehran University Medical Sciences, Tehran, Iran.*; 6 * Education of Development Centre (EDC Department), Medical School, Jahrom University Medical Science, Jahrom, Iran.*

**Keywords:** *Factor structure*, *General Health Questionnaire-28*, *Infertile women*

## Abstract

**Background:**

Nowadays, infertility problems have become a social concern, and are associated with multiple psychological and social problems. Also, it affects the interpersonal communication between the individual, familial, and social characteristics. Since women are exposed to stressors of physical, mental, social factors, and treatment of infertility, providing a psychometric screening tool is necessary for disorders of this group.

**Objective:**

The aim of this study was to determine the factor structure of the general health questionnaire-28 to discover mental disorders in infertile women.

**Materials and Methods:**

In this study, 220 infertile women undergoing treatment of infertility were selected from the Yazd Research and Clinical Center for Infertility with convenience sampling in 2011. After completing the general health questionnaire by the project manager, validity and, reliability of the questionnaire were calculated by confirmatory factor structure and Cronbach's alpha, respectively.

**Results:**

Four factors, including anxiety and insomnia, social dysfunction, depression, and physical symptoms were extracted from the factor structure. 50.12% of the total variance was explained by four factors. The reliability coefficient of the questionnaire was obtained 0.90.

**Conclusion:**

Analysis of the factor structure and reliability of General Health Questionnaire-28 showed that it is suitable as a screening instrument for assessing general health of infertile women.

## Introduction

Approximately, 12% of couples in their reproductive age are infertile (1) and this problem has become a social concern, and is associated with multiple psychological and social problems in today’s world (2). Infertility is considered as a distressing experience, which can lead to different psychological reactions, such as depression, loss of sexual desire, anger, guilt, anxiety, and hopelessness (3-4). However, with the advancement of science and technology and the invention of modern techniques such as intrauterine insemination (IUI), in vitro fertilization (IVF), micro-injection, and other therapeutic methods, hope is increased for infertile couples (5). The results of studies show that women who are undergoing IVF treatment, due to the uncertainty of infertility treatment success, often become depressed and anxious (6). 

Due to psychological-mental problems in infertile women, determining their mental health is essential. General Health Questionnaire (GHQ) is one of the best tools for screening and determining the problems. The original form of GHQ has 60 questions and some forms have been shortened to 12-28 questions. In comparison with the other questionnaires, the 28-item version has the highest level of reliability, sensitivity and specificity (7). This questionnaire has been translated into different languages and its reliability, validity and factor structure have been checked in different cultures. Many studies have been confirmed the 4 components of the GHQ-28 questionnaire using factor structure (8-10). The order of the extracted components and the questions of each component are the only differences observed in different populations. For example, Aderbighe *et al* have surveyed the factor structure in a sample of pregnant women in Nigeria and have confirmed the previous four components (11). 

Several studies have shown that the components of the questionnaire are strongly influenced by the culture and structure of different societies. For example, Taghavi *et al *in a study on students in Shiraz university used factor analysis of public health and derived the four factors of depression, anxiety, social dysfunction, and physical symptoms with the the Cronbach alpha of 0.90. Also, 50.8% of the total variance was explained by these four components (12). Simillarly Molavi *et al* in a study on students in Isfahan University extracted the four components and the reliability of the questionnaire was 0.91 (13). In another study conducted by Ebrahimi *et al* on patients with psychiatric disorders, four factors were derived and the Cronbach alpha was 0.97 (14). In the study of Sadjjadi *et al, *the factor structure of the GHQ in the patients with traumatic brain injury was studied and the reliability coefficient was 0.89. The four extracted factors were anxiety and insomnia, social dysfunction, depression, and physical symptoms (15). In a study by Ghanbarnejad and his colleagues on dermatologic patients, reliability and validity of GHQ-28 were evaluated through examining four different methods (16). Javanmard *et al* assessed the students in East Azerbaijan by the standardization GHQ-28 questionnaire. Four components were extracted from the factor structure, including physical symptoms, anxiety and sleep disorders, social dysfunction and depression, respectively. The reliability was reported 0.87 (17). 

In studies on infertile women, only one GHQ was used to determine mental disorders and none of them have examined the factor structure analysis. For example, Behjati Ardakani *et al* (18), Shakeri *et al* (2) and Baghiani Moghadm *et al *(19) used only one questionnaire to evaluate the psychometric properties and determine the psychological signs on infertility, too . So far, no study has been done using CHQ-28 factor analysis for screening mental disorders in infertile women. Therefore, due to different results of different target groups, perception of infertile women about general health questions, and the difference between extracted components of different populations, this study aimed to address the following questions: 1) Are the four main components of CHQ confirmed by infertile women in this study? 2) Is the order of extracted components consistent with the original questionnaire and other studies? These questions can be answered by examining the factor structure and reliability of this questionnaire.

## Materials and methods

This cross sectional study was performed on 220 infertile women undergoing IVF treatment who referred to Yazd Research and Clinical Center for Infertility during 2011. All women with history of psychological disorders were excluded. Sample size was calculated with a confidence level of 95%, a prevalence of 44% (2), and accuracy of 6.6% *(n=Z*^2^* P(1-P)/d*^2 ^*)*. In this study, the GHQ with 28 questions was used, which were introduced by Hillier and Goldberg in 1979 (7). The questionnaire consists of four sub-components that measure public health (somatic symptoms, anxiety and insomnia, social dysfunction, and depression), and each component consists of 7 questions. GHQ scoring methods are based on the Likert scale from zero to three and a lower score indicates a better mental state. Minimun, maximum, and cut-off score is 0, 84, and 24, respectively. 

The study proposal was approved by Ethics Committee of Jahrom University, Jahrom, Iran. Also, All the participants were informed primarily about the goals and methods of conducting research and after receiving their consent, they filled the questionnaires. Thus, before visiting the doctor and in the waiting time, people filled out the forms. For those who did not have the necessary education or were not able to record due to visual impairments, the questionnaires were read for them and their responses were recorded by the assistant. Individuals with previous history of psychiatric disorders were excluded. By conducting a pilot study on 30 infertile women, Content and face validity and reliability was evaluated Cronbach Alpha was determined 0.89. 

**Statistical methods**

The confirmatory factor analysis was used to evaluate the structural validity of the questionnaire. It was used to test whether the data fit its consistent with a hypothesized model based on the previous research. Two indicators were used: KMO (Kaiser-Meyer-Olkin) for adequacy of the sample size, and Bartlett test for appropriate correlations between variables to do a factor analysis. The reliability of the questionnaire and its components were calculated using Cronbach alpha coefficient. The Pearson correlation coefficient was used to determine the correlation between the components and the total score of the questionnaire. Data analysis was performed using Statistical Package for the Social Sciences, version 16.0, SPSS Inc, Chicago, Illinois, USA (SPSS software). 

## Results

Cronbach alpha employed for estimating reliability before conducting factor analysis. Questions with Cronbach alpha coefficient of less than 0.40 considered to be removed. But, the least value of Cronbach alpha for deleting all items were 0.89. Thus, none of the questions were deleted (Table I).

**Factor analysis**

The KOM value was 0.87 that is higher than 0.8, and showing that the sample size is suitable for factor analysis. Also, the Bartlett test result was significant (p< 0.001), it shows a good correlation between variables for factor analysis. Confirmatory factor analysis with principal component analysis showed that 50.12% of the total variance was explained by four factors. 

These four factors, include anxiety and insomnia, social dysfunction, depression, and physical symptoms. Additionally, the first component accounted for 28.67% of the variance and the fourth component accounted for 4.36% of the variance (Table II). 

Table III shows the method of direct oblimin of the rotated component matrix. As seen, questions related to depression and social dysfunction were in the appropriate dimensions, as expected. 

Question 1 about the physical symptoms was placed in social dysfunction, question 4 in physical symptoms, and questions 3 and 6 changed from anxiety to depression symptoms. 

**Table I:** Reliability statistics of the items of the GHQ-28

**Item-total statistics**					
**Name of scale**s	**Questions**	**Scale mean if item deleted**	**Scale variance if item deleted**	**Corrected item-total correlation**	**Cronbach alpha if item deleted**
A1	1	22.24	111.713	0.362	0.898
A2	2	22.33	114.118	0.239	0.901
A3	3	22.20	109.157	0.573	0.894
A4	4	22.38	109.712	0.547	0.894
A5	5	22.29	113.322	0.293	0.900
A6	6	22.61	111.948	0.386	0.898
A7	7	22.10	110.675	0.441	0.897
B1	8	22.04	107.853	0.542	0.894
B2	9	22.09	109.381	0.485	0.896
B3	10	22.25	112.143	0.347	0.899
B4	11	22.01	107.934	0.623	0.893
B5	12	22.32	107.769	0.637	0.892
B6	13	22.51	108.908	0.568	0.894
B7	14	21.87	108.383	0.537	0.894
C1	15	22.25	113.664	0.388	0.897
C2	16	22.11	116.153	0.211	0.900
C3	17	22.23	112.724	0.442	0.896
C4	18	22.26	113.556	0.427	0.897
C5	19	22.34	114.844	0.324	0.898
C6	20	22.24	112.962	0.451	0.896
C7	21	22.17	111.934	0.456	0.896
D1	22	22.73	108.818	0.620	0.893
D2	23	22.64	109.490	0.548	0.894
D3	24	22.79	109.413	0.608	0.893
D4	25	23.02	114.126	0.398	0.897
D5	26	22.60	109.744	0.555	0.894
D6	27	22.88	109.431	0.649	0.893
D7	28	23.04	114.102	0.477	0.896

GHQ-28: General Health Questionnaire-28

Items A: physical symptoms (questions 1-7), B: anxiety and insomnia (questions 8-14), C: social dysfunction (questions 15-21), D: depression (questions 22-28).

**Table II:** Total variance explained

**Component**	**Rotation sums of squared loadings**	
	**% of variance**	**Cumulative %**
1	28.67	28.67
2	8.70	37.37
3	8.12	45.49
4	4.63	50.12

**Table III:** Factor loading of the items of the GHQ-28 by direct Oblimin rotation

**Name of scale**s	**Questions**	**Component**			
		**1**	**2**	**3**	**4**
B1	8	0.735			
B4	11	0.728			
B7	14	0.724			
B2	9	0.701			
B5	12	0.681			
C3	17		0.773		
C4	18		0.766		
C5	19		0.754		
C1	15		0.587		
C2	16		0.573		
C6	20		0.551		
C7	21		0.496		
A1	1		0.469		
D7	27			0.850	
D3	24			0.835	
D1	22			0.793	
D2	23			0.752	
D7	28			0.733	
D4	25			0.693	
D5	26			0.617	
A4	4			0.521	
B6	13			0.516	
B3	10			0.372	
A6	6				0.806
A5	5				0.748
A7	7				0.528
A3	3				0.504
A2	2				0.466

GHQ-28: General Health Questionnaire-28

Items A: physical symptoms (questions 1-7), B: anxiety and insomnia (questions 8-14), C: social dysfunction (questions 15-21), D: depression (questions 22-28).

**Reliability**

The results of Cronbach alpha coefficients were obtained for; depression, anxiety and insomnia, social dysfunction and physical symptoms 0.90, 0.88, 0.81, 0.78 and 0.69, respectively that demonstrated the internal consistency of the questions (Table IV).

**Correlation**

Internal consistency calculated by the correlation of various dimensions with total scores and the correlation of various dimensions together (Table V). As seen, the lowest correlation was between the components of physical symptoms and social dysfunction (r= 0.287), and the highest correlation was between the components of depression and anxiety (r= 0.566). The correlation between different dimensions and the total score was more than 0.68.

Changes in the correlation of every question with the total scores ranged from 0.265 to 0.704 that is a sign of internal correlation of the questions. The lowest correlation was related to question 2 of social dysfunction and the highest correlation was related to question 6 of depression.

**Table IV:** Cronbach's alpha coefficients of the subscales of GHQ-28

	**Cronbach's alpha coefficients**
Depression	0.88
Anxiety and insomnia	0.81
Social dysfunction	0.78
Physical symptoms	0.69
Total score	0.90

GHQ-28: General Health Questionnaire-28

**Table V:** Inter-correlations between the GHQ-28 subscales and total scale in the infertile women

	**Depression**	**Anxiety and insomnia**	**Social dysfunction**	**Physical symptoms**	**Total score**
Depression	1.000*				
Anxiety and insomnia	0.566	1.000			
Social dysfunction	0.466	0.419	1.000		
Physical symptoms	0.421	0.553	0.287	1.000	
Total score	0.859	0.810	0.700	0.681	1.000

GHQ-28: General Health Questionnaire-28

* Correlation coefficient Note: p< 0.001 for all correlation coefficients

## Discussion

The aim of this study was to examine the factor structure of the CHQ-28 in infertile women. To investigate the reliability of the questionnaire, Cronbach alpha coefficient and to assess the structure validity, factor analysis were used. Previous studies in Iran reported that the validity and reliability of GHQ questionnaire are very high. Taghavi (12), Molavi (13), Yaghoubi (20), and Palahang (21) reported the internal consistency of, 90%, 91%, 88% and 91%, respectively. Also, other questionnaire psychometric aspects were reported and the suitability was confirmed (12-13).

KMO and Bartlett test indicators showed the adequacy of the sample size and appropriateness of the factor analysis. Four factors, including anxiety and insomnia, social dysfunction, depression, and physical symptoms were extracted, respectively. Anxiety and sleep disorders showed 28.67 of the total variance, it is indicative of the importance of this factor in the overall health of infertile women. The four factors extracted in this study were similar to those extracted by foreign and Iranian studies, but the order of their extraction is different. This is partly because of the nature of the samples. Because the role of cultural factors leading to the factor structure is not similar to the other samples. The order of extracted components is similar in studies of Javanmard (17), Ebrahimi (14), Molavi (13), and Gibbons (9). In none of the studies, the extracted components order were not similar to our study. For this reason, the importance of the factor analysis of the GHQ became clear in infertile women.

The Cronbach alpha value indicated the internal consistency of the questionnaire. In the present study, Cronbach's alpha was higher than 0.75 for all of the components except for the physical symptoms, indicating that the questionnaire has the internal consistency reasonably in Iranian infertile women. 

It is consistent with the studies of Rezaei (15), Molavi (13), and Malakouti (22). High value of Cronbach's alpha of depression component means the suitability of the questions to determine the depression in infertile women and low value of Cronbach's alpha of physical symptoms indicates that personal evaluation of infertile women was difficult to physical symptoms.

The results indicated a significant correlation between the four extracted components. Anxiety and depression showed the highest correlation, it is consistent with the studies of Javanmard (17) and Rezaei (15). Questions of social dysfunction and depression are loaded correctly in corresponding component in agreement with the original version of the questionnaire. Other studies also have shown that these two components are reasonably stable in different populations (9, 10, 23). 

It seems that infertility treatment affects women’ health, so that women under treating show high levels of anxiety, depression and emotional stress (24); incidence of depression and anxiety is more than in women who have failed to IVF, and 10 to 25 percent of women are at risk of depression and anxiety (25). Therefore, using a suitable tool could contribute to the treatment of infertile women in the diagnosis of mental disorders.

It is recommended that clinical findings should be used to determine the psychometric disorders in addition to general health screening test. 

While using the data only from infertile women is a limitation of our study, it is recommended to use the data about the spouses of infertile women.

## Conclusion

The structure validity and internal consistency of GHQ-28 and compatibility with the results of other studies showed that these questionnaires are appropriate, understandable and clear for infertile women, and they have not been faced with problem in the research. 

Although the extracted factors are different from other studies, the results of the factor structure will approve this questionnaire for the psychiatric disorders’s study in infertile women.
